# Gap Junctional Communication via Connexin43 between Purkinje Fibers and Working Myocytes Explains the Epicardial Activation Pattern in the Postnatal Mouse Left Ventricle

**DOI:** 10.3390/ijms22052475

**Published:** 2021-03-01

**Authors:** Veronika Olejnickova, Matej Kocka, Alena Kvasilova, Hana Kolesova, Adam Dziacky, Tom Gidor, Lihi Gidor, Barbora Sankova, Martina Gregorovicova, Robert G. Gourdie, David Sedmera

**Affiliations:** 1 Institute of Anatomy, First Faculty of Medicine, Charles University, 128 00 Prague, Czech Republic; veronika.olejnickova@fgu.cas.cz (V.O.); kocka.matej@centrum.cz (M.K.); alena.kvasilova@lf1.cuni.cz (A.K.); hkole@lf1.cuni.cz (H.K.); adam.dziacky@gmail.com (A.D.); tomgidor@gmail.com (T.G.); lifshitzlihi@gmail.com (L.G.); bara.sankova@centrum.cz (B.S.); martina.greg@email.cz (M.G.); 2 Institute of Physiology, CAS, 142 20 Prague, Czech Republic; 3 Department of Pediatric Cardiology, Motol University Hospital, 150 06 Prague, Czech Republic; 4 Fralin Biomedical Research Institute, Virginia Tech, Roanoke, VA 24016, USA; gourdier@vtc.vt.edu

**Keywords:** connexin, cardiac conduction system, optical mapping, myocardium, immunohistochemistry

## Abstract

The mammalian ventricular myocardium forms a functional syncytium due to flow of electrical current mediated in part by gap junctions localized within intercalated disks. The connexin (Cx) subunit of gap junctions have direct and indirect roles in conduction of electrical impulse from the cardiac pacemaker via the cardiac conduction system (CCS) to working myocytes. Cx43 is the dominant isoform in these channels. We have studied the distribution of Cx43 junctions between the CCS and working myocytes in a transgenic mouse model, which had the His-Purkinje portion of the CCS labeled with green fluorescence protein. The highest number of such connections was found in a region about one-third of ventricular length above the apex, and it correlated with the peak proportion of Purkinje fibers (PFs) to the ventricular myocardium. At this location, on the septal surface of the left ventricle, the insulated left bundle branch split into the uninsulated network of PFs that continued to the free wall anteriorly and posteriorly. The second peak of PF abundance was present in the ventricular apex. Epicardial activation maps correspondingly placed the site of the first activation in the apical region, while some hearts presented more highly located breakthrough sites. Taken together, these results increase our understanding of the physiological pattern of ventricular activation and its morphological underpinning through detailed CCS anatomy and distribution of its gap junctional coupling to the working myocardium.

## 1. Introduction

The mammalian ventricular myocardium consists of cardiac myocytes, which are thought to form a functional syncytium due to gap junctional channels (GJC). Each GJC is comprised of connexins (Cxs), which are, in general, important for metabolic and electric communication between the adjacent cells (reviewed in [1]). In the heart, Cxs have both direct and indirect roles in the transmission of the electrical impulse from the cardiac pacemaker via the cardiac conduction system (CCS) to the working myocytes [2,3]. Conduction properties of myocardial GJC depends on particular connexin isoforms and their assembly into homotypic, heterotypic or heteromeric channels [4,5,6]. The function of the specific motifs of connexins in the regulation of the formation of GJC in the heart was reviewed in [7].

In the mammalian atrial and ventricular myocardium, Cx43 represents the dominant isoform. Its conduction properties could by modified by post-translational modifications, mostly by de/phosphorylation [8,9,10]. Today, 21 phosphorylation sites are described for Cx43 and it is thought that all stages of the Cx43 “life cycle” are modified by phosphorylation [11]. Moreover, many pathophysiological conditions, such as ischemia [12,13], hemodynamic overload [14,15] and diabetes [16,17] may affect Cx43 phosphorylation and thus change vulnerability to arrhythmias [10]. Apart from alterations in phosphorylation, also impaired Cx43 expression, spatial distribution, and heterogeneity are considered to have a pro-arrhythmic potential [18,19]. Moreover, the role of Cx43 hemichannels in pro-arrhythmic environment was recently revied in [20]. Similarly, the alteration in Cx40 is associated with certain type of arrhythmias [21,22].

In contrast to working myocardium, the Purkinje myocytes in the terminal part of the CCS express Cx40, as well as Cx43 [23,24]. In addition, the entire CCS also expresses Cx45 [25,26], the first connexin to appear during cardiogenesis [27], which continues to be expressed throughout the myocardium in small amounts including the nodes [26], where Cx30 is also detected [28]. Later in development appears Cx40, which is a marker of chamber myocardium differentiation and is said to allow for faster transmission of the electrical impulses [29,30]. The abundant expression of Cx43 in the chamber myocardium is unique to mammals [31] and may be an adaptation to enable fine and rapid regulations [14,32,33].

The existence of Purkinje-working myocardium coupling is traditionally described only in homoiotherms, as no evidence for the Purkinje fibers (PFs) was found in the poikilotherms [34,35]. The adult heart achieves the Purkinje-working myocardium junctional contacts through Cx43. On the other hand, during development small amounts of Cx40 are present also in the compact myocardium [36,37], making Cx40 Purkinje-myocyte coupling a possibility during the embryonic and early postnatal stages. These junctions, as well as PFs in general, can be a source of arrhythmias [38,39].

Studying the activation sequence of the ventricular myocardium in three dimensions is a challenge. From the electrocardiogram it is deduced that the transmural activation proceeds from the endocardium (containing the PFs) to the epicardium. Epicardial optical mapping shows apex-to-base activation with the first activated regions located near ventricular apices [37,40,41], corresponding to bundle branches’ terminations. Transmural activation is clearly dependent on intramural PFs in the hearts where those are present, e.g., in Ungulates [38]. However, neither the human, nor the mouse has intramural PFs [42], and little hard data is available on the actual nature of physiological propagation of the transmural activation wave [43,44,45,46].

Many cartoons representing the CCS do not respect the true anatomy of the ventricular conduction fascicles. First, the bundle branches are flat and especially the left one resembles little a cable-like structure [36,47]; second, they do not in fact even reach the apex of the heart, breaking into a mesh of PFs after coursing about 2/3 down the interventricular septum. Lastly, human (and mouse) hearts do not have intramural PFs, whereas Ungulates [36,47,48] or avians display a considerable number of these morphologically distinct cells [49,50]. These important facts need to be considered when constructing a realistic functional 3D model of the conduction pathway in the heart that would correspond with the real data [51].

The overarching goal of our research is to improve our understanding through providing data on detailed conduction system structure and function in the mouse heart, which closely resembles the human one from the point of CCS anatomy. This paper focuses on the most peripheral CCS component, the PFs, and their terminations in (or connection with) the working ventricular myocardium. To elucidate the anatomy and physiology of the CCS components, we applied a combined morphological (immunoconfocal microscopy) and functional (optical mapping of intact hearts) approach to study the nature and frequency of Purkinje-myocyte junctions and epicardial pattern of ventricular activation. In parallel, we studied the morphological maturation of the PFs in relation to the working cardiomyocytes (shape characteristics, nuclear count). We hypothesized that the amount of PFs and their connections with the working left ventricular myocardium would reflect the typical observed apex-to-base activation of the left ventricle.

## 2. Results

### 2.1. Cx43 Expression in Purkinje-Myocyte Junctions

**Figure 1:**
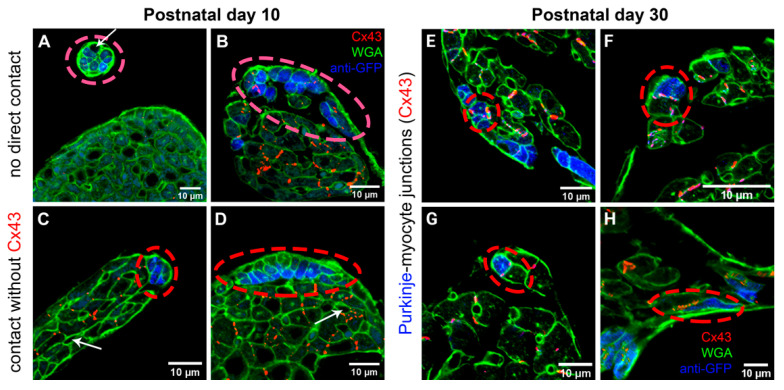
Illustration of Purkinje-working myocytes connections in the mouse heart. At P10, no Cx43-positive coupling between the Purkinje (blue) and working (green outlines) myocytes were detected (**A**–**D**). White arrows indicated capillaries among the myocytes. Areas of contact with Cx43-positive gap junctional coupling (red dots) observed at P30 (**E**–**H**) were still much less frequent than cases without such connection. Note also increased width of the working myocytes between these stages.

Serial sectioning of the hearts at postnatal day 1 (P1) showed still extensive GFP-positive (i.e., Cx40-expressing) trabecular network, which, while expressing also abundantly Cx43, did not show any Cx43 connections with the compact myocardium (data not shown). It was impossible to distinguish the future PFs from the rest of the myocardial trabeculae based solely upon GFP expression in these Cx40-GFP mouse hearts. Similarly, no such connections were detected at P10, but the GFP expression was now clearly restricted to the subendocardial Purkinje network (Figure 1). A total of 63 sites with Cx43-based Purkinje-myocyte connections in the left ventricle were found in one of the hearts sectioned transversely at 20 microns and imaged entirely at P30 (Figure 1).

**Figure 2:**
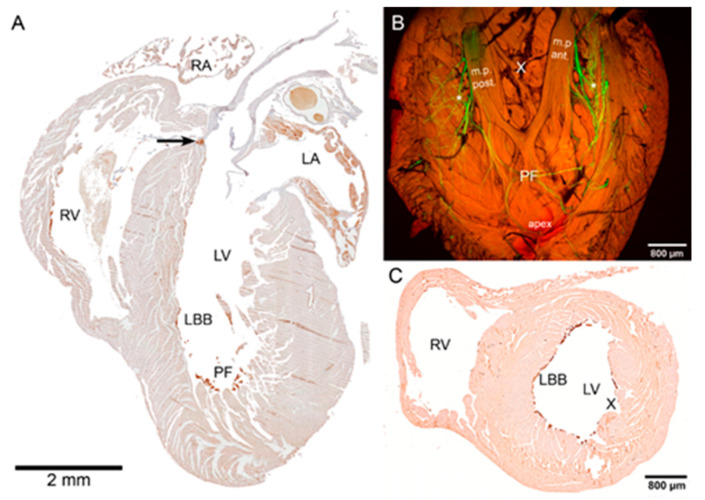
Distribution of PFs within the P30 left ventricle. (**A**) Four-chamber view shows part of the His bundle at the crest of the interventricular septum (arrow) and part of the left bundle branch (LBB), with a high abundance of the Purkinje fibers in the apical area (PF). (**B**) Whole mount confocal projection (maximum intensity, 32 optical sections, z-step 50 micrometers) of left ventricular free wall using 2× objective shows both the apical PF cluster and then the two ascending strands (*) along the papillary muscles. The area between the papillary muscles (corresponding externally to the obtuse margin of the heart) is conspicuously devoid of PFs (X), as documented also on the transverse section (**C**). Anti-GFP immunostaining with diaminobenzidine as a color substrate (**A**,**C**), native GFP fluorescence (green) with autofluorescence (red) on an optically cleared specimen (**B**); LA, left atrium, LV, left ventricle, m.p. ant & post, anterior and posterior papillary muscles, RA, right atrium, RV, right ventricle.

**Figure 3:**
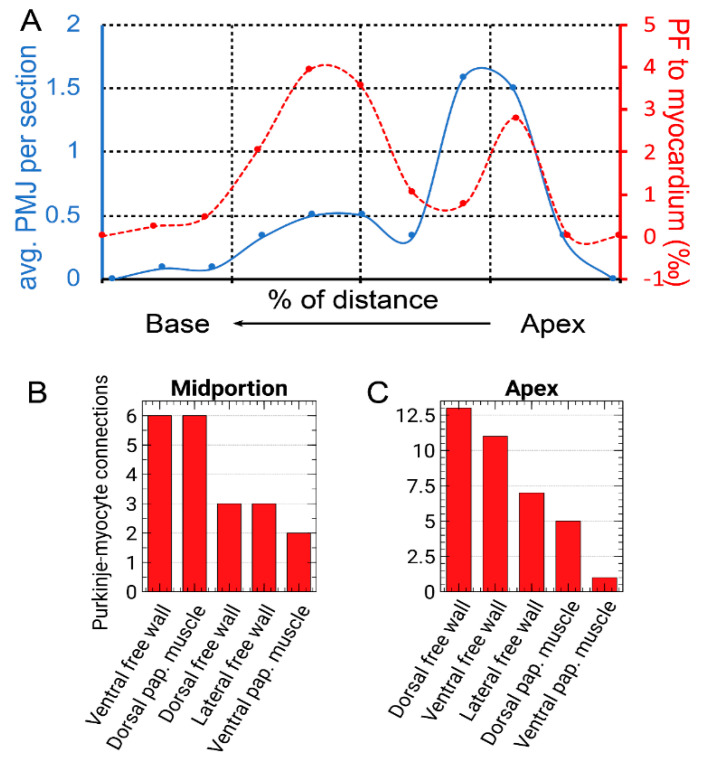
(**A**) Quantification of PF distribution (normalized as a proportion to working myocardium, red dashed line) and Purkinje-myocyte connections (average number per a cross-section, from 4 consecutive 20-micrometer sections spanning 80 µm, blue solid line) along the apico-basal axis. Data from two representative hearts (red and blue circular markers) are normalized on the same x-axis as a percentage of apico-basal distance. Note a peri-apical peak around 25% of the distance, detected by both methods (compare with Figure 2), and a second peak at around 60% distance towards the base. Panels (**B**,**C**) show the number of Purkinje-myocyte connections at different segments of the left ventricular circumference at the mid-ventricular and apical area, respectively, corresponding approximately to the peaks in panel (**A**).

To quantify the amount of PFs by normalization to the amount of the working myocardium, transverse serial sectioning and visible staining were used (Figure 2). It showed the highest amount of PFs at 60% of the apex-base distance, with a second peak in distribution around the ventricular apex (±20%, Figure 3) with almost no PFs at the base. The distribution of Cx43-positive connections between the Purkinje and working myocytes showed a peak in abundance between 20–30% of the distance from ventricular apex to the base, corresponding to the region with the highest PF to working myocardium ratio. The paucity of the PFs in the basal area was further validated by whole mount imaging of the dissected endocardial surface of the left ventricle (Figure 2).

The distribution of PFs around the ventricular perimeter was also non-random. From the apex, there were two major ascending pathways on the left ventricular free wall, one located dorsally and one ventrally, ascending along the papillary muscles (Figure 2). This perception was also confirmed by quantification of the Purkinje-myocyte connections proportion in different ventricular segments in the apical and midportion region. The highest proportion was found correspondingly on the dorsal and ventral free wall, with lower numbers on the ventral papillary muscle and a lateral free wall (between the papillary muscles, Figure 3).

The Purkinje network was, with the exception of the apical luminal region, flat against the ventricular endocardial surface and thus possible to image in 3D (Figure 2) when the heart was dissected and pinned flat with the endocardial surface up. The network formed ellipsoidal shapes with bundles few cells thick connecting and diverging with virtually no free ends.

### 2.2. Epicardial Activation Patterns of the Left Ventricle

**Figure 4:**
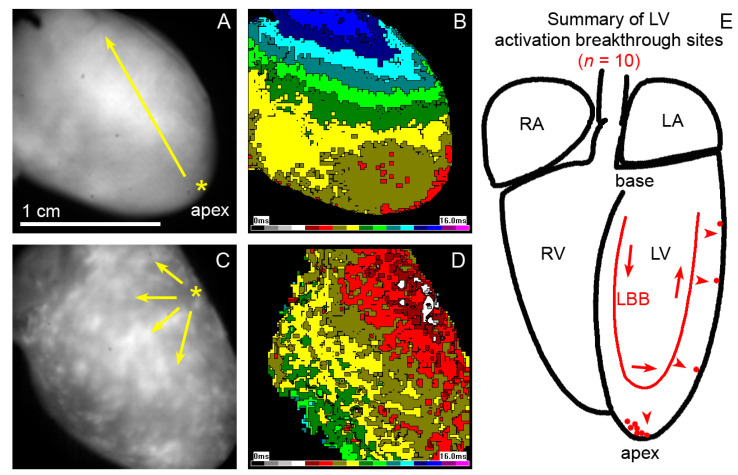
Epicardial activation maps of the 1-year-old mouse hearts showing a typical apical breakthrough (**A**,**B**) and a less frequent one (**C**,**D**) from a higher position on the left ventricular free wall (yellow asterisks). Yellow arrows indicate an apparent direction of activation wave spreading across the left ventricular surface. The cartoon on the right (**E**) shows a summary from 10 hearts (each red dot representing a single heart) aged between 12–15 months. Red arrows indicate the impulse traveling through the conduction system, red arrowheads the transmural spread. LA, left atrium, LBB, left bundle branch, LV, left ventricle, RA, right atrium, RV, right ventricle.

To evaluate functional consequences of the observed patterns of PFs connections to the working myocardium, epicardial optical mapping was performed on 10 normal mouse hearts aged 12–15 months (Figure 4). In the majority of the hearts, the left ventricle was activated from the apex; however, in 30% of cases, the first breakthrough was located at some distance from the apex along the left (obtuse) margin of the heart.

### 2.3. Cellular Analysis of the PF Network

**Figure 5:**
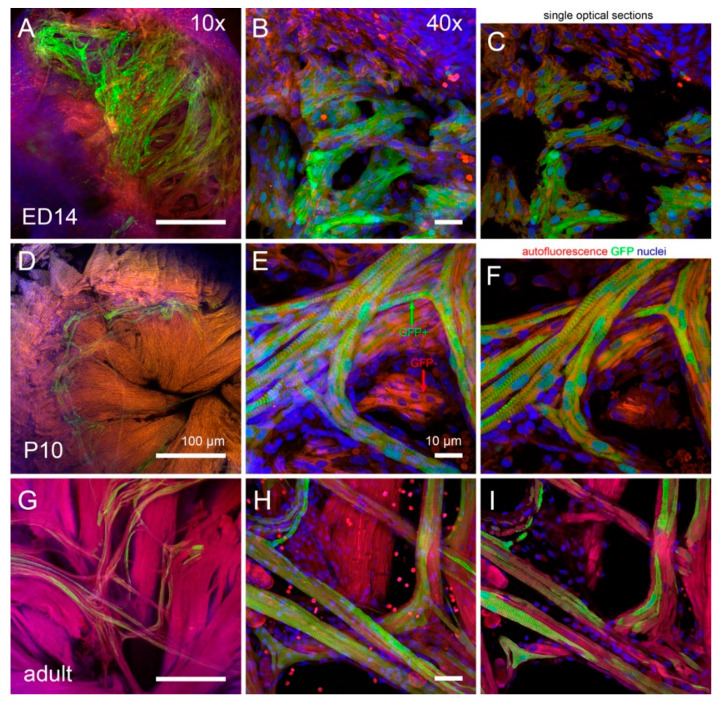
Mid- and high-resolution imaging of fetal (ED14, **A**–**C**) and postnatal (P10, **D**–**F**; adult, **G**–**I**) mouse hearts for nuclear counts and cell dimensions show a clear increase in cell size between these stages, especially thickening of the working myocytes and elongation of the Purkinje myocytes. Low magnification (**A**,**D**,**G**) was used to select a region of interest for higher power imaging. Panels (**B**,**E**,**H**) are maximum intensity projections of the high-resolution images taken with 40× oil immersion lens and were used for making sure that the whole cell is included, while the diameters were measured on single optical sections (**C**,**F**,**I**). Note that the GFP-positive Purkinje network (green arrow) is clearly distinguishable from the remaining GFP-negative (red arrow) ventricular trabeculations (trabeculae carnae) by P10. In all cases, the left ventricular apex is shown.

Cell shape analysis was performed in whole mount hearts at embryonic day (ED) 14 (end of septation), P10 and adult (6 months, Figure 5). At the ED14, the GFP-positive cells of the trabeculae were distinct from the compact layer, but their size was similar and both populations were primarily mononucleated and still in the proliferative phase [52].

**Figure 6:**
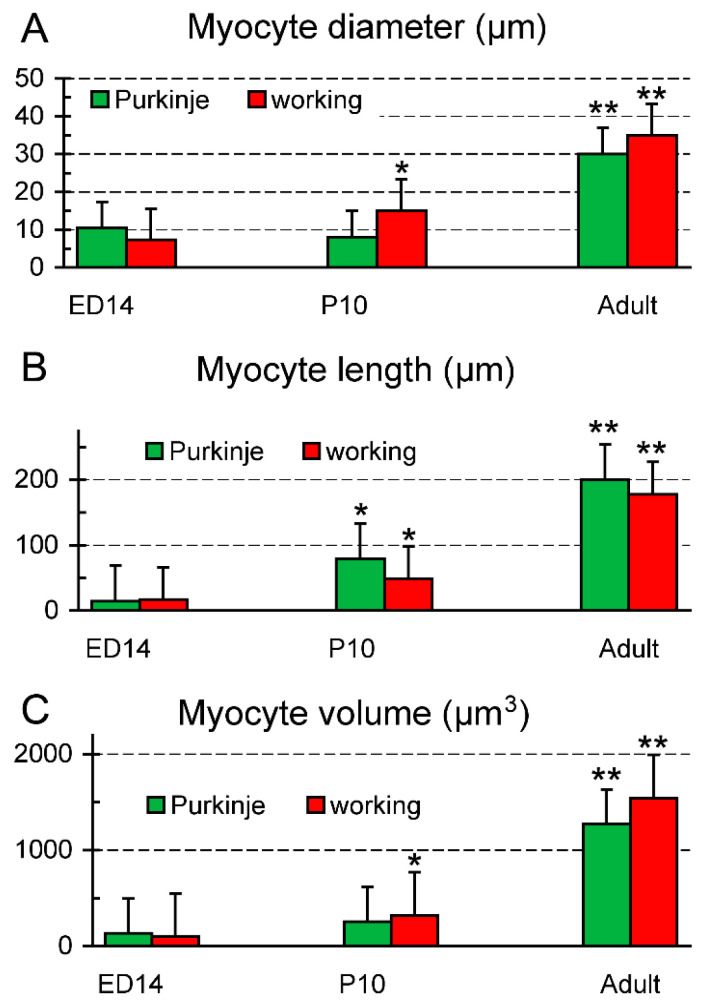
Development of shape characteristics of the Purkinje and working myocytes. (**A**) transverse diameter (width) across the nucleus, (**B**) the longest diameter measured in 3D on the confocal stack, (**C**) calculated cell volume (assuming cylindrical geometry). The Purkinje myocytes and notably thinner and more elongated than their working counterparts. Data are mean ± SD, * *p* < 0.05 between P10 and ED14, ** *p* < 0.05 between the previous stages, assessed by unpaired two-tailed *t*-test.

While at P10 the working myocytes showed a clear increase in transverse diameter (Figure 1 and Figure 5), the width of the GFP-positive PFs was essentially unchanged, so the comparable increase in cell volume was due mostly to their elongation (Figure 6). This resulted in a significant change in length to width ratio (from 1.4 vs. 2.3 at ED14 to 9.9 vs. 3.3 at P10 for the PFs and working myocytes, respectively; *t*-test, *p* < 0.05 between the working and Purkinje myocytes at P10). In the adult, there was a further increase in volume in both groups, due to both cell thickening and elongation, resulting in a length to width ratio of 6.7 and 5.1, respectively.

**Figure 7:**
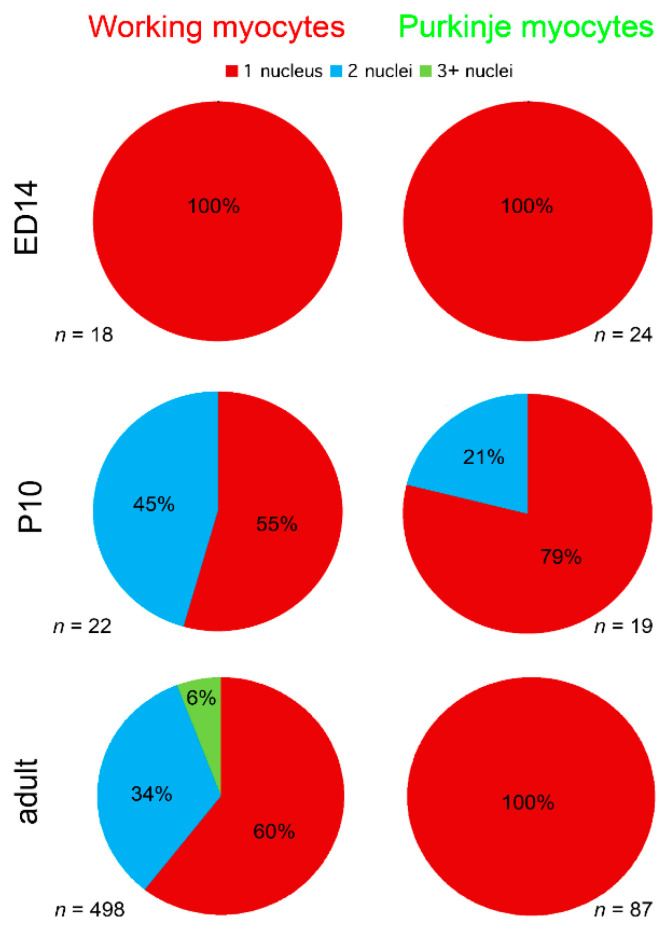
Development of nuclear counts in murine working and Purkinje myocytes. At the prenatal stage (ED14), all myocytes are mononucleated. Binucleation appears at P10. Summary analysis of nuclear counts from 3 adult (6 months of age) hearts (bottom row) shows that while the average proportion of the mononucleated working myocytes was 60%, all the analyzed Purkinje myocytes possessed only a single nucleus.

The analysis of cell nuclear count showed that all the myocytes were strictly mononucleated at ED14, while at P10, some working, as well as a few of the Purkinje myocytes showed two nuclei. In the adult, however, while around 40% of the working myocytes had more than one nucleus, all 87 Purkinje myocytes analyzed in 3D contained a single nucleus (Figure 7).

## 3. Discussion

### 3.1. Maturation of Cx43 Expression

In agreement with previous studies [53], Cx43 organization in the ventricular myocardium proceeds during postnatal development from almost isotropic to strongly aligned expression at the cell ends with fewer lateral connections. It correlates with increasing conduction velocity, as demonstrated by optical mapping of the right atrium in rabbits [54]. Cx43 was detected in both the compact layer and trabeculae at fetal and postnatal stages, however, from our data it appears that it participates in Purkinje-myocyte gap junctional coupling only past P10, as we could not find any Cx43-based Purkinje-myocyte connections prior to that date despite abundant Cx43 expression in the trabeculae. Presumably, the connections until then are dependent on Cx40, which is initially expressed also in the embryonic left ventricular compact zone [37,55]. The working and conduction myocytes are derived from common precursors, as demonstrated by progressive restriction of the Cx40-expressing cells to the conduction fate in the chick [49] and mouse [56], which, together with a rather long half-life of Cx40 protein and Cx45 expression could also explain the maintenance of electrical impulse propagation in Cx43 null newborn mice [57]. Similarly, Cx43 (and also Cx45) are compensating in the settings of Cx40 deficiency to maintain the conduction in the atria [30]; thus, the largest decrease in the conduction velocity was noted at the early stages before Cx43 expression sets on, while at the later ones, the differences became much less pronounced. However, due to different half-life and isoform switching in different myocyte populations during development the picture is rather complex. The conductive differences observed in the embryonic heart (acceleration of conduction, establishing of the preferential pathway [37,58] could well be in the background of this change during development). This notion is corroborated by recent findings of common clonal origin of PFs and working myocytes, with precursors Cx40-expressing precursors committing to these distinct lineages shortly before birth under Nkx2.5 control [52]. Thus, quantification of these connections could only be performed in the adult mice using the Cx40:eGFP transgene in heterozygous state as a bona fide PF marker. Using 20-micron thick sections and high-resolution confocal microscopy greatly facilitated complete sampling of the contacts between the Purkinje network and working myocardium, which are not too frequent, akin to holes in hoses of a drip irrigation system.

### 3.2. Distribution of Purkinje-Myocyte Junctions and Its Correlation with the Ventricular Activation Pattern

The highest density of Cx43 connections between the PFs and working myocytes was observed at 20–30% of the distance between the left ventricular apex and the base. At this distance, on the septal surface of the left ventricle, the insulated left bundle branch splits into the network of (uninsulated) PFs [36]. This point is also regarded as the origin of the septal electrical activation [51,59]. Thus, the wavefront does not have to reach first the apical endocardial aspect of the left ventricular free wall, as the PFs in the apical area are located in the ventricular lumen that they cross to the lateral free wall (Figure 2). This explains correlation of the peaks in the proportion of PFs and their connections at one-third of the apex-base distance (Figure 3). The lack of correlation in the more apical area is likely due to the fact that those traversing fibers (Figure 2) serve mostly as a conduit from the septum and do not form many connections with the working myocardium. The observation of the first epicardial activation in the apical region in a majority of hearts (Figure 4) is not at all contrary to these results, since the transmural propagation, likely to be rather uniform in the absence of the intramural PFs [46,51], is influenced by the distance traveled, i.e., the thickness of the ventricular wall. This is considerably thinner in the apical compared to the basal area, as illustrated in various species [60,61], and the difference is apparent already from the prenatal period [62,63].

### 3.3. Morphological Characteristics of the Purkinje Network

At the macroscopic (whole ventricle) level, imaging using the 2× objective showed the general pattern of distribution with numerous, highly GFP-positive bundles in the apex and ascending strands along the anterior and posterior papillary muscles (Figure 2). This uneven distribution with a notable paucity of fibers on the free wall between the papillary muscles was confirmed also on transverse sections and is similar to the original description of Tawara in Ungulates, dogs and humans [47]. A higher level of detail could be appreciated on 2D maximum intensity projections (using confocal imaging with 10× objective and z-step between 3 and 10 micrometers), but the true 3D arrangement with the possibility of delineation of individual cells was only achieved with 25× and higher magnification objective lenses (Figure 1 and Figure 5). Nuclear co-staining allowed establishing the nucleation level; while the level of cells with more than one nucleus for the working myocytes in the adult hearts was between 35 and 45% (Figure 7), the adult Purkinje myocytes were strictly mononucleated, in contrast to those of the Ungulates [42,48]. This may enable them to re-enter the cell cycle should it be necessary during heart regeneration after injury in the adulthood, as the mononucleated state is deemed to be a pre-requisite for proliferation [64]. The relatively higher proportion of binucleated myocytes (both working and conducting, Figure 7) at P10 could be explained by the gradually increasing cell cycle length at the end of the proliferative phase of development, which increased the temporal separation between the karyokinesis and cytokinesis [65,66]. We also found that postnatal Purkinje myocytes were thinner and more elongated compared to the working myocytes (Figure 6). Their network was closed with virtually no free ends, forming ellipsoidal spaces [36,52]. The bundles were formed by 2–4 GFP-positive cells thick bundles (Figure 1 and Figure 5) and their closed apposition made the exact delineation of cell borders in whole mount challenging. Our parallel examination of their network in the right ventricle did not detect any gross difference in the pattern, apart from generally smaller density compared to the left ventricle.

### 3.4. Conclusions

We found the highest number of connections between PFs and cardiomyocytes in the periapical region, where the left bundle branch splits to form the Purkinje network, with a second peak of PF number in the apex. Most of the adult mouse left ventricles were indeed activated from the apex, but there were some higher activation sites, reminiscent of the earlier developmental stages. The differentiating Purkinje myocytes started to show Cx43 connections with the working myocytes after postnatal day 10 and increased their volume chiefly by elongation starting by day 10. Interestingly, they remained in the adulthood, in contrast to their working counterparts, mononucleated.

### 3.5. Limitations, Future Directions, Perspectives

Our study of the connections is based on serial sections, possibly missing some of the connections that could be detected in whole mount [67]. Our methodological approach is limited to the adult (1 month and older) stages, since prior to that, the Purkinje-myocyte junctions seem to be dependent on Cx40 or Cx45 gap junctional coupling. While other PF markers such as contactin-2 [68] were reported, we concentrated on the Cx40:GFP transgenic model as it enabled simple whole mount imaging after clearing [55] without any additional staining thanks to its strong cytoplasmic expression.

It would be interesting to provide direct measurements of transmural activation during spontaneous rhythm at different locations and compare those with the patterns from animals with developed intramural Purkinje network, such as sheep [38]. This would be especially useful for extrapolation from the data from these large mammalian models to the human situation [46], which is from this perspective more similar to the mouse.

Finally, recent data suggest a role for ephaptic conduction of activation in mammalian working ventricular myocardium [69]. Interestingly, the evidence suggests that gap junctions may have a non-canonical role in facilitating close intercellular contacts required for such electric field-based mechanisms of cardiac conduction to operate. Future work might also address whether ephaptic mechanisms also operate in the CCS and/or at PF-myocyte junctions [70].

## 4. Materials and Methods

### 4.1. Heart Sampling and Processing

Cx40:GFP mouse hearts [36] in heterozygous state were used for this study. The mice were kept in controlled environment in compliance with all applicable regulations with food and water ad libitum. The wild-type females were caged overnight with Cx40:GFP homozygous males of proven fertility, and in the morning checked for vaginal plugs; the noon of the day of plug discovery was considered ED0.5. The pregnant females were killed at ED14.5 by cervical dislocation, and the litters were rapidly dissected in ice-cold phosphate buffer saline (PBS). The embryos were then quickly decapitated, and the hearts were perfused with 4% paraformaldehyde in PBS, followed by immersion in the same fixative for 24 h. The hearts were then rinsed with PBS and the nuclei were counterstained with Hoechst 333422 nuclear stain (Sigma #861405, Darmstadt, Germany; 1:40,000 in PBS with 0.5% Triton-X). The samples were then cleared with Scale/2 as described [55]. The hearts were then mounted into Scale in cavity slides, coverslipped, and carefully sealed with nail polish for whole-mount imaging on a Leica SP8 inverted confocal microscope using 10× dry or 40× oil immersion objectives. The stacks were collected with z-step of 10 micrometers (10× lens) or 1 micrometer (40× lens).

The hearts from heterozygous animals killed by cervical dislocation at P10 and six months of age (P180, adults) were treated in the same fashion, except that the ventricles had to be microdissected into smaller pieces (apex, left and right ventricular free wall) to enable mounting (Figure 5). Three hearts per stage were analyzed.

Whole mount confocal imaging without nuclear counterstaining (Figure 2) was performed on P30 hearts (*n* = 6) after their dissection, pinning and clearing using CUBIC [55] (whole PF network imaging, Figure 2) on a confocal microscope (Olympus BX61, FluoView) using 2× (NA 0.14), 4× (NA 0.16), 10× (NA 0.4), or 25× dipping (NA 1.0) objectives.

### 4.2. Quantitative Analysis

Quantitative analysis of myocyte dimensions (Figure 6) and nuclear counts (Figure 7) were performed by a blinded observer on 3D confocal stacks. The transverse diameter of both working (red, Figure 5) and conductive (green) myocytes was measured on single optical sections running through the nucleus. For this purpose, the individual cell images were extracted from the stacks and measured using caliper tool in Adobe Photoshop 8.0 (Adobe Systems, Palo Alto, CA, USA). Myocyte length was measured only on myocytes that were captured entirely in the stack in 3D using ImageJ. Only on such completely captured cells were also performed nuclear counts (Figure 7).

For analysis of Purkinje-myocytes connections, three postnatal hearts at P1, P10 (end of proliferative phase) [71], and P30 (weaning) were perfusion-fixed with 4% solution of paraformaldehyde, embedded in paraffin blocks, and cut in series at 20 microns in a short-axis (two chamber) plane. The sections were then de-paraffinized, rehydrated with descending ethanol series, blocked in 10% normal goat serum in phosphate buffer saline with 0.5% Triton-X, and stained sequentially with anti-Cx43 (1:200; Sigma #C6219, Darmstadt, Germany) and anti-GFP (1:500; Abcam #6556, Cambridge, UK) with heat inactivation (10 min in 50% glycerol in PBS at 100 °C) between the steps, as both primary antibodies were developed in rabbit. Anti-Cx43 was detected with Cy5-coupled goat-anti-rabbit secondary (1:200; Jackson ImmunoResearch #111-175-144), and anti-GFP was detected with TRITC-coupled goat-anti-rabbit secondary (1:200; Jackson ImmunoResearch #111-025-144, Ely, UK). Cell borders were visualized with Alexa488-conjugated wheat germ agglutinin (WGA, Invitrogen #W11261, ThermoFisher Scientific, Waltham, MA, USA). Imaging was performed on an Olympus Fluoview 1000 confocal microscope using 60× oil immersion lens (NA 1.42) with z-steps ranging from 0.5 to 2 micrometers, so that the area of interest was captured entirely. All Cx43-positive connections found were documented as z-stacks (Figure 1). The counts were binned from four consecutive sections spanning 80 micrometers and plotted after normalization as a percentage of the distance between the apex and the base. The Purkinje-myocyte connections thus found were also assigned to the following locations: ventral and dorsal free wall, ventral and dorsal papillary muscle, and the area between them (Figure 3).

For validation purposes, additional hearts (three per stage) were cut at 10 microns in the transverse plane and every fourth section was stained for anti-GFP detected with diaminobenzidine for quantification of the ratio of PFs to working myocardium (Figure 2). Quantification of the ratio of GFP-positive PFs to the GFP-negative working myocardium was also performed in the adults and plotted after normalization to percentages. Sister sections were stained with Cx43/GFP/WGA/Hoechst combination to verify the distribution of Purkinje-myocyte connections. To complement the 3D conceptualization of PFs arrangement, three more hearts per stage were cut in the coronal plane. Imaging was performed using the 10× objective on an Olympus BX51 microscope with an Olympus DP80 CCD camera.

### 4.3. Optical Mapping

Optical mapping of left ventricular epicardial activation pattern was performed with the Ultima L CMOS camera on normal adult mouse hearts (*n* = 10, 12–15 months) using either 2× objective (NA 0.14) on an Olympus BX51 FS upright microscope (12 months), or a BrainVision macroscope (www.scimedia.com (1 March 2021)) with Leica objectives (15 months). The isolated hearts were perfused in a horizontal Langendorff chamber (Radnoti, Inc., Covina, CA, USA) and bolus-stained with di-4-ANEPPS (Invitrogen #D1199) as described [72]. Activation maps in sinus rhythm were generated using the bundled software BV_ANA as described [37] (Figure 4).
